# Adult age-group differences in domain-specific environmental appraisal under standardized exposure

**DOI:** 10.3389/fpsyg.2026.1838100

**Published:** 2026-09-11

**Authors:** Lingyu Kong, Dongkwon Seong

**Affiliations:** Department of X-Cultural Studies, Graduate School of Kookmin University, Seoul, Republic of Korea

**Keywords:** adult age differences, comparative judgment, domain-specific appraisal, environmental appraisal, perceived environmental support, person-environment fit, standardized exposure

## Abstract

Adult age-group differences in environmental judgment may be expressed through domain-specific differences in comparative appraisal under standardized exposure conditions. Drawing on environmental appraisal theory, this study examined whether perceived environmental support is organized as a domain-specific comparative appraisal process and whether age group moderates the distribution of such appraisal across domains under standardized exposure conditions. Using a within-subject comparative rating design, 804 adults from South Korea and China evaluated two thematically matched cultural exhibition settings presented through standardized visual materials. Perceived environmental support was assessed using 17 study-specific appraisal items aggregated into five theory-guided domains for domain-level comparative analysis. Results from the full-sample analysis showed significant Context × Domain and Context × Domain × Age Group interactions in linear mixed-effects models, indicating that comparative judgments followed a domain-differentiated pattern and that this domain profile varied across age groups rather than reflecting a uniform preference for one context. The overall domain-differentiated pattern was highly stable under repeated age-balanced subsampling, whereas the age-moderation effect showed lower resampling stability. Age-group profiles showed no simple monotonic progression across adulthood. Together, these findings support a domain-distributed account of environmental appraisal in which age-group variation is located in the configuration of support-relevant appraisal rather than overall evaluative tendency.

## 1 Introduction

Physical environments influence judgment not only through their material properties but also through how they are psychologically appraised (Mehrabian and Russell, 1974; Bitner, 1992; Kotler, 1973; Tam and Milfont, 2020). Environmental psychology has accordingly emphasized appraisal, perceived control, and affective–cognitive processing as key processes shaping human responses to settings (Mehrabian and Russell, 1974; Bitner, 1992). In person–environment research, a setting is supportive when its demands are compatible with users’ capacities and it enables effective action (Iwarsson and Ståhl, 2003; World Health Organization [WHO], 2007). Perceived environmental support is therefore better understood as a multidimensional psychological appraisal than as a single overall impression or a direct indicator of physical quality (Iwarsson and Ståhl, 2003; World Health Organization [WHO], 2007; Bonaiuto et al., 1999; Blaison and Hess, 2016).

Age is theoretically relevant in this process because environmental appraisal may vary across adulthood, particularly when judgment depends on legibility, navigation-related information, accessibility, and confidence in use (Lester et al., 2017; Fricke et al., 2022). Research on aging and navigation shows that older adults often experience greater difficulty in unfamiliar or visually complex settings, especially when successful movement depends on spatial updating, route integration, and the interpretation of environmental information (Lester et al., 2017; Bosch and Gharaveis, 2017). Wayfinding studies further indicate that layout complexity, signage, route alignment, visual access, and pathway configuration shape navigation performance, including among older adults (O’Neill, 1991; Passini, 1981; Weisman, 1981; Wiener et al., 2009; Arthur and Passini, 1992; Peponis et al., 1990; Merhav and Fisher-Gewirtzman, 2023; Gath-Morad et al., 2021; Gath-Morad et al., 2024; Zuo and Zhou, 2024).

Age-friendly environment research likewise highlights legibility, accessibility, safety, and confidence in use as central conditions of later-life support (World Health Organization [WHO], 2007). More broadly, environmental gerontology suggests that support in later life emerges from dynamic person–environment exchange involving usability, control, belonging, and environmental affordances, rather than from physical accessibility alone (Oswald et al., 2006; Wahl and Oswald, 2016; Chaudhury and Oswald, 2019; König et al., 2019; Wahl et al., 2009). Taken together, these findings suggest that circulation, information, lighting, service usability, accessibility, and safety may not contribute equally to environmental appraisal across age groups; more generally, legibility and coherence remain central dimensions in environmental appraisal and preference (Scopelliti and Giuliani, 2004; Merhav and Fisher-Gewirtzman, 2023; Zuo and Zhou, 2024; Stamps, 2004).

Despite this broader theoretical basis, relatively little research has examined whether age-related differences in environmental judgment are expressed simply as a more favorable or less favorable overall evaluation, or instead as domain-specific differences in the relative prominence of support-relevant appraisal under standardized exposure conditions. This distinction matters because environmental support is unlikely to operate as a single undifferentiated quality across adulthood. Rather, prior work on wayfinding, legibility, age-friendly environments, and person–environment exchange suggests that circulation, information, lighting, service usability, accessibility, and safety may differ in their relative prominence within environmental appraisal across adult age groups (Lester et al., 2017; Fricke et al., 2022; Oswald et al., 2006; Chaudhury and Oswald, 2019; König et al., 2019; Wahl et al., 2009; Scopelliti and Giuliani, 2004; Stamps, 2004). The present study therefore tests whether comparative environmental appraisal is domain-structured and whether its profile varies across adult age groups under standardized exposure.

By standardizing the exposure format and comparing judgments within the same respondents, the design allows domain-specific appraisal profiles to be compared under matched cue conditions. The two stimulus sets served as matched environmental contexts for examining how adults of different ages distribute comparative judgments across distinct channels of perceived environmental support, consistent with research linking spatial configuration and accessibility to environmental experience and use (Wineman and Peponis, 2010; Psarra and Grajewski, 2000; Koustriava and Koutsmani, 2023).

Building on this theoretical foundation, the present analysis evaluates two non-directional hypotheses. First, if perceived environmental support is organized as a domain-differentiated appraisal process rather than as a single global evaluation, the comparative effect of stimulus context should vary across appraisal domains. Accordingly, H1 proposes that comparative appraisal will show a significant Context × Domain interaction. Second, prior research on navigation, environmental legibility, and person–environment exchange suggests that the relative prominence of support-relevant appraisal domains may differ across adult age groups. Accordingly, H2 proposes that the domain-specific comparative pattern will vary across age groups, reflected in a significant Context × Domain × Age Group interaction. These hypotheses are intentionally non-directional with respect to specific domains and age groups because the present analysis tests whether appraisal is distributed differently across domains and age groups rather than assuming a universal hierarchy of environmental preferences.

## 2 Materials and methods

### 2.1 Study design and stimulus materials

This study used a standardized within-subject comparative rating design in which the same respondents evaluated two public-environment stimulus sets as sources of perceived environmental support. The design follows environmental-appraisal research treating physical settings as psychologically active conditions that shape appraisal, perceived control, and evaluative response (Mehrabian and Russell, 1974; Bitner, 1992). The present secondary analysis focuses on two thematically matched focal settings selected from the multiple museum settings rated in the original questionnaire.

At the beginning of the questionnaire, respondents received brief introductory information defining the relevant museum and spatial-design context. During the rating task, appraisal items were presented within the corresponding setting-specific presentation structure, together with visual stimulus materials where available, and the same respondents evaluated both focal contexts under matched presentation conditions. When a corresponding environmental feature was absent from a focal setting, no separate visual example of that feature was presented for that item–setting combination. The resulting scores were interpreted as comparative appraisal judgments under standardized exposure, with matched presentation conditions maintained across the two focal contexts (Kong, 2025).

Table 1 summarizes the source-context traceability of the two stimulus contexts. The original item-specific visual presentations are documented in Supplementary Tables S1, S2, including item–setting combinations in which the corresponding feature was absent from the focal setting and therefore no separate visual example was presented. The full original questionnaire, including item wording, response categories, and the item-specific visual presentation format, is reproduced in Appendix of the publicly available master’s thesis from which the present secondary-analysis data were drawn (Kong, 2025).

**TABLE 1:** Source-context traceability for the two standardized exposure sets.

Country	Context code	Broad setting type
South Korea	Context B	Themed cultural exhibition setting
China	Context A	Themed cultural exhibition setting

Context codes are used throughout the analysis. Full visual stimulus materials are reproduced in Supplementary Tables S1, S2, and the full original questionnaire is available in Kong (2025).

### 2.2 Participants, recruitment, and procedure

The present study reports a secondary analysis of an anonymous online survey conducted between March 7 and April 25, 2024, as part of a master’s thesis at Chonnam National University (Kong, 2025). Of the 1,105 questionnaires initially collected, 214 were excluded during the original data-screening process because of missing data, internally inconsistent responses, or clearly patterned responding, leaving 891 valid responses. The present secondary analysis further excluded 87 respondents younger than 20 years, yielding a final adult sample of 804 valid responses from South Korea and China.

Age was collected in the original questionnaire using prespecified decade-based categorical response options. After excluding respondents younger than 20 years, the retained adult categories were 20–29, 30–39, 40–49, 50–59, and 60 years or older. The present secondary analysis therefore retained these original survey categories rather than constructing age groups post hoc from continuous age data. The resulting age-group sizes were unequal because they reflect the respondent distribution in the original survey. The primary analysis retained all eligible adult respondents without balancing or subsampling, and no additional recruitment was undertaken. Demographic characteristics are presented in Table 2.

**TABLE 2:** Sample characteristics.

Characteristic	N	%
Country		
South Korea	587	73.0
China	217	27.0
Gender		
Female	448	55.7
Male	356	44.3
Age group		
20–29	258	32.1
30–39	147	18.3
40–49	210	26.1
50–59	120	14.9
60+	69	8.6

Respondents completed a within-subject rating task in which the same appraisal items were presented with corresponding stimulus materials for multiple museum settings. The present secondary analysis retained the 17 internal-space appraisal items for two thematically matched focal contexts, one from South Korea and one from China. The same respondents rated both focal contexts using the same five-category response format and item-specific stimulus presentation structure.

### 2.3 Study-specific domain composites for comparative appraisal

Perceived environmental support was assessed using 17 study-specific items grouped a priori into five theory-guided appraisal domains: circulation and spatial ease, hierarchical information organization, illumination and visual atmosphere, maintenance and service usability, and environmental access and safety cues. The items were developed for the original survey as study-specific indicators and were not adapted verbatim from a single established psychometric scale. Their grouping and interpretation in the present analysis were informed by prior work on person–environment fit and accessibility, environmental appraisal, wayfinding and legibility, and age-supportive environmental conditions (Iwarsson and Ståhl, 2003; World Health Organization [WHO], 2007; Bitner, 1992; Passini, 1981; Weisman, 1981; Oswald et al., 2006).

Domain means were specified a priori as composite indices for repeated comparative analysis under standardized exposure conditions. Because the five domains were theoretically specified rather than empirically derived, exploratory factor analysis was not used for post hoc scale construction; confirmatory factor analysis was used as a context-specific diagnostic of the prespecified grouping. Substantive inference was conducted at the domain-composite level, with measurement diagnostics used to evaluate the interpretability of that aggregation (Kline, 2023; Vandenberg and Lance, 2000; Putnick and Bornstein, 2016; Luong and Flake, 2023). Responses were recorded on a five-category response scale ranging from the most favorable to the least favorable response and were reverse-coded for analysis (original 1–5 responses transformed as 6 - response), so that higher analytic scores indicated more favorable appraisal. The domain definitions are summarized in Table 3, and the full item list is provided in Table 4.

**TABLE 3:** Five theory-guided appraisal domains used for domain-level comparative analysis.

Appraisal domain	Appraisal focus
C. Circulation and spatial ease	Perceived support for entry, movement, rest, and spatial ease
H. Hierarchical information organization	Perceived support for organizational clarity, display clarity, and viewing appropriateness
I. Illumination and visual atmosphere	Perceived support from lighting adequacy and visual readability
M. Maintenance and service usability	Perceived support from signage, service access, and facility usability
E. Environmental access and safety cues	Environmental support from visible or communicated access- and safety-enabling cues

The five domains are theory-guided, study-specific composites used for domain-level comparative analysis under standardized exposure conditions.

**TABLE 4:** Study-specific stimulus-bound indicators used to derive domain-level comparative appraisal scores.

Appraisal domain	Perceived support indicator	Item code	Analytic item description
C. Circulation and spatial ease	Perceived entry support	Q1	The entry area appears to provide adequate and clearly legible space for comfortable passage.
	Circulation continuity	Q2	The main circulation paths appear level and free of steps or abrupt level changes.
	Movement clarity	Q3	Circulation in the main viewing area is clear and appears to support smooth movement.
	Rest support	Q4	The rest area appears to provide adequate space for pausing or resting.
	Browsing-related spatial support	Q5	The retail or browsing area appears to support comfortable browsing and movement.
H. Hierarchical information organization	Interpretive ordering	Q6	The presented materials appear to be organized in a logical and coherent order.
	Display clarity support	Q7	Display structures appear to support clear viewing of the presented materials.
	Viewing appropriateness	Q8	The spacing and viewing height of the display elements appear appropriate.
I. Illumination and visual atmosphere	Directional lighting support	Q9	Lighting appears to be positioned and directed appropriately.
	Illumination adequacy	Q10	Lighting appears to provide adequate and reasonably even illumination.
	Visual detail support	Q11	Overall lighting quality appears to support clear perception of visual details and material features.
M. Maintenance and service usability	Wayfinding information support	Q12	Information and directional signage appear visible and easy to interpret.
	Service accessibility	Q13	The main service point appears easy to identify and accessible to use.
	Facility usability and privacy	Q14	Essential service facilities appear clean, clearly organized, and usable.
E. Environmental access and safety cues	Enabling step-free access cues	Q15	Visible access features appear to support use by people with reduced mobility.
	Enabling accessibility information cues	Q16	Supportive information for navigation or access appears available where needed.
	Environmental safety-support cues	Q17	Visible environmental features appear to support safe use, including for more vulnerable users.

These stimulus-bound indicators were developed for the original survey and grouped into theory-guided appraisal domains for the present comparative analysis. The English descriptions summarize the content of the original questionnaire items for the present domain-level analysis; the complete original item wording and presentation format are available in Kong (2025).

### 2.4 Statistical analysis

Statistical analyses were conducted to examine domain-specific comparative appraisals of perceived environmental support under a repeated-measures structure in which the same respondents evaluated both stimulus contexts. Because ratings were nested within respondents, the primary analyses used linear mixed-effects models (LMMs) with respondent-specific random intercepts and Context slopes to account for within-person dependence in repeated ratings (Yu et al., 2022; Bates et al., 2015; Barr et al., 2013).

Consistent with H1 and H2, the main inferential focus was the Context × Domain interaction, followed by the Context × Domain × Age Group interaction, because these terms directly tested whether comparative appraisal patterns differed across the five domains and whether those patterns varied across adult age groups. Likelihood-ratio tests for the key interactions are reported in Table 5, and planned within-domain contrasts are reported in Table 6. When multiple related contrasts were interpreted jointly, Benjamini–Hochberg false discovery rate correction was applied (Benjamini and Hochberg, 1995).

**TABLE 5:** Likelihood-ratio tests comparing primary mixed-effects models.

Comparison	LL (full)	LL (reduced)	LR χ ^2^	df	*p*
Context × domain interaction (score model)	−7100.891	−7145.709	89.637	4	< 0.001
Context × domain × age group interaction (score model)	−6983.546	−7004.412	41.731	16	< 0.001

**TABLE 6:** Planned within-domain comparative appraisal contrasts (Context B-Context A).

Domain	Δ (B-A)	CI low	CI high	dz	*p*	p(FDR)
C	−0.082	−0.133	−0.031	−0.167	0.002	0.002
H	−0.164	−0.215	−0.113	−0.221	<0.001	<0.001
I	−0.067	−0.118	−0.016	−0.092	0.010	0.010
M	−0.187	−0.238	−0.135	−0.258	<0.001	<0.001
E	0.122	0.071	0.173	0.188	<0.001	<0.001

All planned contrasts were coded as Context B-Context A. Negative values indicate higher ratings for Context A, whereas positive values indicate higher ratings for Context B. Cohen’s dz values represent within-subject effect sizes. p (FDR) denotes Benjamini–Hochberg false discovery rate-adjusted p values.

Although responses were recorded using five-category response scales, the main analyses were conducted on aggregated domain mean scores because the primary aim was to evaluate domain-level comparative appraisal patterns rather than item-level latent structure. Measurement diagnostics evaluated the interpretability of domain aggregation, while substantive inference was conducted at the domain level. Given the unequal age-group sizes, age-stratified follow-up contrasts were interpreted with emphasis on the estimated pattern and magnitude of contrasts rather than on the number of statistically significant domains within each subgroup. As an additional sensitivity analysis addressing the unequal country composition of the sample, the primary models were re-estimated with participant country included as a between-subject fixed-effect covariate. Country was entered as a main effect only, while the original random-effects structure was retained. No Country interaction terms were introduced because the purpose of this analysis was adjustment for sample composition rather than testing cross-national moderation.

To examine the robustness of the findings under an age-balanced reduced sample, we conducted a repeated age-balanced subsampling sensitivity analysis. In each of 200 iterations, 69 respondents were sampled without replacement from each of the five age groups, corresponding to the size of the smallest 60+ group and yielding *N* = 345 per iteration. All repeated observations from selected respondents were retained, and the primary mixed-effects model specification was refitted unchanged. We summarized the proportion of iterations in which the Context × Domain and Context × Domain × Age Group interactions remained significant, the median and interquartile range of the likelihood-ratio statistics, and the directional stability of the overall within-domain contrasts.

### 2.5 Ethics statement

The original data were collected through an anonymous online survey conducted between March 7 and April 25, 2024, as part of the first author’s master’s thesis project at Chonnam National University. Participation was voluntary, and electronic informed consent was obtained from all respondents before access to the questionnaire was granted. No personally identifiable information was collected or recorded in the dataset. The present study is a secondary analysis of a fully de-identified dataset and involved no new recruitment, participant re-contact, intervention, or linkage to identifiable information. In accordance with local legislation and institutional requirements applicable to anonymous, non-interventional questionnaire research and secondary analysis of de-identified data, formal IRB review was not required.

## 3 Results

### 3.1 Sample characteristics

A total of 1,105 questionnaires were initially collected. After restricting the dataset to adult respondents and applying the study’s screening procedures, the final analytic sample comprised 804 valid responses. As shown in Table 2, 73.0% of respondents were from South Korea and 27.0% from China; 55.7% were female and 44.3% male. The age distribution was 32.1% for 20–29 years, 18.3% for 30–39 years, 26.1% for 40–49 years, 14.9% for 50–59 years, and 8.6% for 60 years and above.

### 3.2 Descriptive internal-consistency checks for domain aggregation

Table 7 reports Cronbach’s α and McDonald’s ω for each theory-guided domain by context and for the overall 17-item set. The overall item set showed acceptable to good internal consistency in both contexts (Context A: α = 0.897, ω = 0.900; Context B: α = 0.869, ω = 0.883), while several three-item domains showed more modest coefficients, consistent with the known sensitivity of internal-consistency estimates to scale length (Cortina, 1993). These coefficients were treated as descriptive checks on domain aggregation.

**TABLE 7:** Descriptive internal-consistency checks for theory-guided domain aggregation by stimulus context.

Scale/Domain	k	Context A		Context B	
		α	ω	α	ω
C. Circulation and spatial ease	5	0.703	0.708	0.732	0.733
H. Hierarchical information organization	3	0.687	0.694	0.673	0.674
I. Illumination and visual atmosphere	3	0.619	0.620	0.692	0.700
M. Maintenance and service usability	3	0.630	0.631	0.637	0.660
E. Environmental access and safety cues	3	0.637	0.677	0.688	0.732
Overall (17 items)	17	0.897	0.900	0.869	0.883

Cronbach’s α and McDonald’s ω are reported as descriptive internal-consistency checks for the study-specific domain composites.

### 3.3 Context-specific structural diagnostics

Table 8 reports context-specific CFA diagnostics for the theory-guided five-domain item grouping estimated with WLSMV for ordinal responses. Fit was acceptable though not ideal for Context A (CFI = 0.949, TLI = 0.936, RMSEA = 0.065, SRMR = 0.049) and weaker for Context B (CFI = 0.866, TLI = 0.833, RMSEA = 0.103, SRMR = 0.078). The CFA was therefore treated as a context-specific structural diagnostic of the prespecified grouping, with substantive inference conducted at the domain-composite level. Descriptive loading congruence across contexts is reported in Supplementary Table S3, and standardized item-level loadings are provided in Supplementary Table S8.

**TABLE 8:** Context-specific CFA diagnostics for the theory-guided five-domain item grouping (WLSMV, ordinal).

Stimulus context	χ ^2^	df	*p*	CFI	TLI	RMSEA	SRMR
Context A	483.164	109	< 0.001	0.949	0.936	0.065	0.049
Context B	1036.210	109	< 0.001	0.866	0.833	0.103	0.078

CFA estimates are reported as context-specific structural diagnostics of the prespecified grouping; substantive inference is conducted at the domain-composite level.

### 3.4 Hypothesis tests in the primary mixed-effects model

Consistent with H1, the primary mixed-effects model showed a significant Context × Domain interaction, χ^2^ (4) = 89.637, *p* < 0.001, indicating that comparative appraisal differed across domains rather than reflecting a uniform context effect. Consistent with H2, the Context × Domain × Age Group interaction was also significant, χ^2^(16) = 41.731, *p* < 0.001, indicating that the domain-differentiated appraisal pattern varied across adult age groups. Both key interaction tests remained significant when participant country was included as an additional fixed-effect covariate: Context × Domain, χ^2^(4) = 89.637, *p* < 0.001, and Context × Domain × Age Group, χ^2^(16) = 41.731, *p* < 0.001. The country-adjusted sensitivity results are reported in Supplementary Table S10.

The repeated age-balanced subsampling analysis showed that the Context × Domain interaction remained significant in all 200 balanced subsamples (100%; median LR χ^2^ = 32.236, IQR = 27.214–36.839). The Context × Domain × Age Group interaction showed lower resampling stability, remaining significant in 101 of 200 subsamples (50.5%; median LR χ^2^ = 26.313, IQR = 21.459–30.251). All five overall within-domain contrasts retained the same direction as the full-sample analysis in 100% of iterations. These results indicate high stability of the overall domain-differentiated pattern but greater sensitivity of the age-moderation effect to reduced and balanced sample composition. Full results are reported in Supplementary Table S11. Approximate random-effects variance components and model R^2^ are reported in Supplementary Table S4. Residual diagnostics for the three-way mixed model are shown in Supplementary Figures S1, S2. As a robustness check, the key interaction effects were also retained in Gaussian GEE analyses (Supplementary Table S5), and the full robust coefficient estimates are provided in Supplementary Table S6.

### 3.5 Planned within-domain contrasts and effect sizes

**FIGURE 1:**
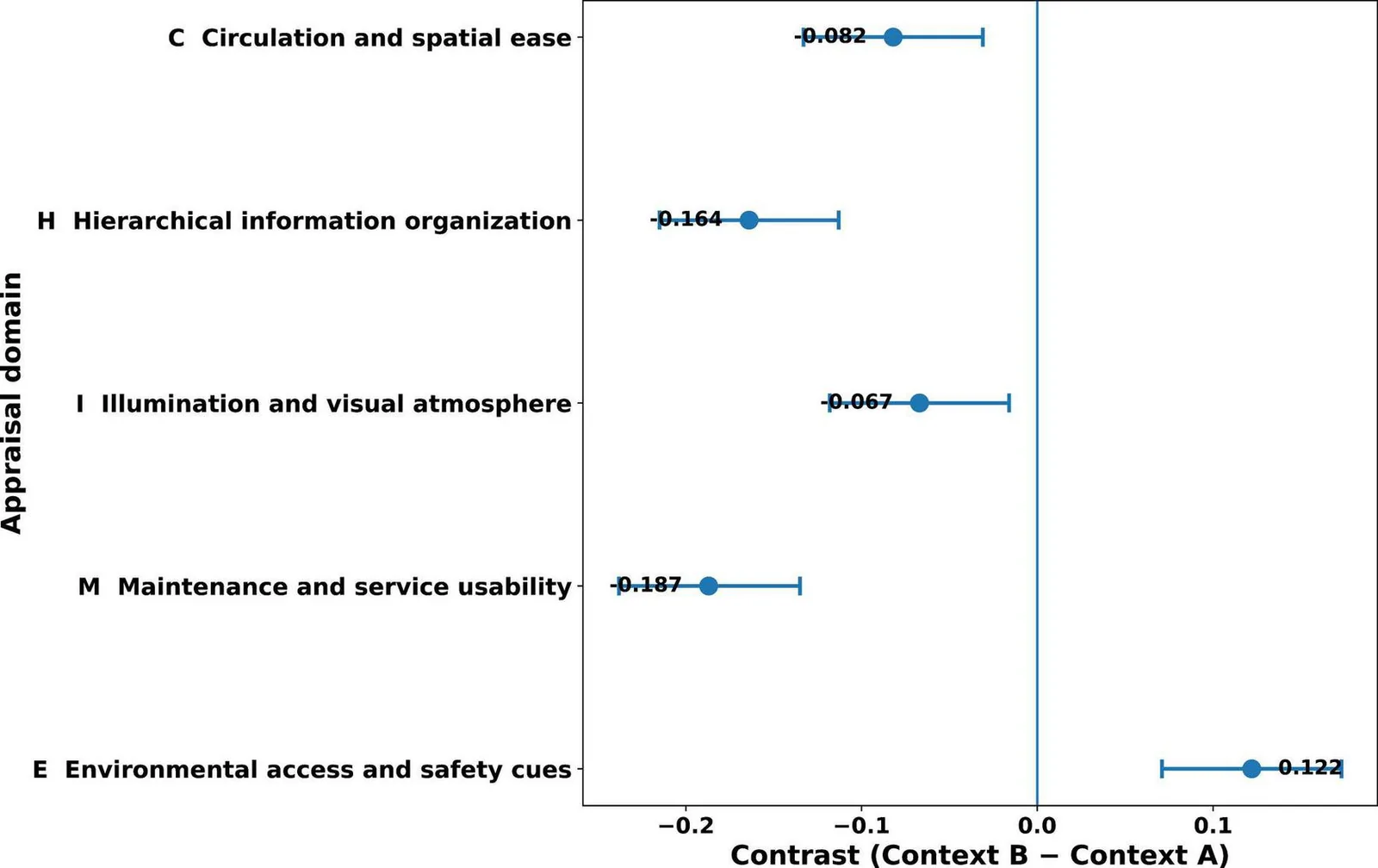
Domain-specific comparative appraisal contrasts with 95% confidence intervals. Points represent model-based contrasts (Δ = Context B - Context A) with 95% confidence intervals. Negative values indicate higher ratings for Context A, whereas positive values indicate higher ratings for Context B.

Planned within-domain contrasts revealed a clear cross-domain reversal in comparative appraisal. As shown in Figure 1 and Table 6, Context A was rated more favorably in circulation and spatial ease, hierarchical information organization, illumination and visual atmosphere, and maintenance and service usability, whereas Context B was rated more favorably in environmental access and safety cues. This reversal indicates a differentiated appraisal profile rather than overall context superiority.

Within-subject effect sizes were modest (| dz| ≈ 0.09–0.26), while the contrast directions formed a consistent cross-domain pattern. The same directional pattern was retained in both the South Korean and Chinese subsamples (Supplementary Table S7).

### 3.6 Age moderation (three-way mixed model)

To examine whether the domain-differentiated appraisal pattern varied across age groups, age-stratified within-domain contrasts were estimated from the three-way mixed-effects model. As shown in Table 9, the 20–29 group showed significant contrasts across all five domains after BH-FDR correction, whereas the remaining age groups showed different combinations of domain-specific contrasts. Because subgroup sizes differed, the number of individually significant contrasts was not interpreted as evidence of progressively broader or narrower appraisal with age. Instead, the full-sample results indicate that age group moderated the distribution of appraisal across domains, with the observed profile varying selectively and non-monotonically across adulthood. The full model-based within-domain contrasts by age group are reported in Supplementary Table S9, and a graphical summary is provided in Supplementary Figure S3.

**TABLE 9:** Age-stratified within-domain comparative appraisal contrasts (Context B - Context A).

Age group	C	H	I	M	E
20–29	−0.135*	−0.252*	−0.112*	−0.337*	0.174*
30–39	−0.004	−0.122	−0.054	−0.027	0.190*
40–49	−0.054	−0.167*	0.033	−0.057	0.029
50–59	−0.045	−0.067	−0.133	−0.167*	0.242*
60+	−0.200	−0.087	−0.116	−0.391*	−0.145

Values are model-based contrasts from the three-way mixed-effects model and were coded as Context B-Context A. Positive values indicate higher ratings for Context B, whereas negative values indicate higher ratings for Context A. Asterisks indicate BH-FDR-adjusted significance.

## 4 Discussion

The results show that comparative environmental appraisal was organized by domain. In the full-sample analysis, this domain profile varied across adult age groups under standardized exposure. The central theoretical implication is that age-group variation appeared in the configuration of support-relevant appraisal rather than as a uniform shift in overall favorability.

### 4.1 Domain-specific appraisal pattern

The cross-domain reversal shows that context effects depended on the appraisal channel: four domains favored Context A, whereas environmental access and safety cues favored Context B. This pattern is consistent with multidimensional accounts of environmental appraisal, in which usability, spatial legibility, atmosphere, and other setting-related cues contribute distinguishable components of environmental judgment (Bitner, 1992; Wineman and Peponis, 2010; Bonaiuto et al., 1999; Blaison and Hess, 2016; Custers and Aarts, 2003; Hanyu, 2000). The observed pattern therefore reflects differentiated psychological appraisal across support-related domains.

### 4.2 Age moderation of domain-specific appraisal profiles

In the full-sample analysis, age group moderated the domain profile of appraisal rather than overall favorability. Age-stratified contrasts differed in configuration and showed no simple monotonic progression across adulthood. The pattern therefore indicates context-dependent age-group variation in the relative prominence of appraisal domains.

Socioemotional selectivity theory provides one plausible interpretive lens for this variation because motivational priorities may shift as perceived time horizons change across adulthood (Carstensen et al., 1999). Because time perspective and motivational goals were not measured, the present data do not test this mechanism directly. The observed age-group variation is also compatible with research showing that navigation and environmental interpretation in later life are sensitive to legibility, spatial configuration, and visual access (Lester et al., 2017; Fricke et al., 2022; Passini, 1981; Weisman, 1981; Merhav and Fisher-Gewirtzman, 2023; Gath-Morad et al., 2021; Gath-Morad et al., 2024).

### 4.3 Measurement and inferential boundaries

The five domains function as study-specific analytical composites for the present comparative analysis. Their short length and uneven item-level fit across contexts limit claims of cross-context item equivalence or a transportable latent measurement model (Kline, 2023; Vandenberg and Lance, 2000; Putnick and Bornstein, 2016; Luong and Flake, 2023). Substantive inference therefore rests on domain-composite patterns rather than latent-scale structure.

Because no direct process or individual-capacity measures were collected, the study cannot determine whether the observed age-group pattern reflects differences in visual acuity, working-memory capacity, processing speed, mobility, attentional allocation, navigation-related interpretation, perceived control, cognitive load, or other unmeasured processes. These remain plausible explanatory factors rather than measured mediators. The cross-sectional age-group comparison also cannot separate biological aging from cohort-related differences in cultural background, accumulated experience, education, or environmental familiarity.

Unequal age-group sizes, particularly the smaller 60+ subgroup, reduced the precision of age-stratified contrasts. Age-balanced subsampling retained the direction of all overall domain contrasts but showed lower stability for the three-way interaction, which remained significant in 50.5% of subsamples. Because balancing reduced the analytic sample from *N* = 804 to *N* = 345, this sensitivity reflects both sample composition and reduced power. The overall domain pattern was therefore more robust than the age-moderation effect, which requires replication in more evenly balanced samples.

Standardized visual exposure does not capture objective environmental performance, actual visitor behavior, or the full multisensory character of in situ experience, including acoustic conditions, dynamic lighting, pedestrian flow, and bodily fatigue. At the same time, the matched within-subject design strengthens inference about comparative appraisal structure because the same respondents evaluated both contexts using the same indicators under standardized exposure. Controlled visual-exposure formats can therefore support focused affective and evaluative comparisons while leaving real-world behavioral performance outside the present inference (Mouratidis and Hassan, 2020).

### 4.4 Psychological implications

From an environmental psychology perspective, the present findings support a profile-based view of environmental appraisal. Comparative judgment was not organized as a single continuum of overall favorability; instead, the two environmental contexts showed different comparative advantages across circulation, informational clarity, visual atmosphere, service usability, and access- and safety-related support. This cross-domain reversal suggests that perceived environmental support is better understood as a configuration of distinguishable appraisal dimensions than as a unitary judgment of environmental quality.

This perspective also gives age-group variation a more specific psychological meaning. Rather than appearing as a uniform increase or decrease in environmental favorability, age-group differences were expressed in how the domain profile of appraisal was configured. The implication is that adult age differences in environmental judgment may concern the relative prominence of different forms of environmental support within the same comparative task. This moves the analytical focus beyond mean-level age differences toward the organization of appraisal itself.

The findings therefore connect environmental appraisal research with person–environment perspectives in environmental gerontology. In these perspectives, environmental support emerges through the relationship between environmental demands, available resources, and the capacities of the individual rather than residing in the setting as a fixed property (Iwarsson and Ståhl, 2003; Oswald et al., 2006; Wahl and Oswald, 2016). The present results add a profile-based dimension to this relational view by showing that perceived support can be differentiated not only in magnitude but also in profile: different support channels can assume different relative prominence across adult age groups even when the environmental stimuli and appraisal domains are held constant.

Accordingly, the contribution of the study is not simply to document age-group differences in environmental ratings, but to position domain configuration as a meaningful level of analysis for understanding person–environment variation across adulthood.

## 5 Conclusion

Comparative environmental judgment was domain-structured, and the full-sample analysis indicated that its profile varied across adult age groups under standardized exposure. The study therefore contributes a profile-based psychological account of age-group variation in environmental appraisal: differences emerged in the configuration of support-relevant domains without a uniform shift in overall favorability. Because the analysis used study-specific composites and standardized visual stimuli, the findings concern domain-level appraisal patterns rather than a general psychometric model or developmental mechanism. More broadly, the results suggest that multiple support channels jointly organize environmental appraisal and may differ in relative prominence across adulthood.

## Data Availability

The original contributions presented in the study are included in the article/Supplementary material, further inquiries can be directed to the corresponding author.
