# Supplementary material for: Adult age-group differences in domain-specific environmental appraisal under standardized exposure
Source: Front Psychol. 2026 Sep 11;17:1838100. doi: 10.3389/fpsyg.2026.1838100 (PMC13612204; doi:10.3389/fpsyg.2026.1838100)
Supplement: Supplementary file 1 [file Supplementary_file_1.DOCX]

This additional file reproduces the standardized visual stimuli presented to respondents in the original comparative rating task and reports supplementary statistical analyses relevant to the main analyses. **Tables S1–S2 reproduce the images used in the original questionnaire for the two exposure conditions and organize them according to the environmental features relevant to the corresponding appraisal judgments.** These materials document the stimulus content used in the comparative rating task and the supplementary analyses supporting the main results.

**Table S1.**

*Visual stimuli presented to respondents for standardized exposure condition B*

| Cue category | Presented feature | Appraisal relevance |
| --- | --- | --- |
| **movement-related cues** | | |
| Entry area |  | Relevant to judgments of entry clarity and ease of passage. |
| Main circulation path |  | Relevant to judgments of route continuity and unobstructed movement. |
| Main viewing area |  | Relevant to judgments of movement clarity within the primary viewing space. |
| Rest area |  | Relevant to judgments of pause/rest support during use of the setting. |
| Retail/browsing area |  | Relevant to judgments of browsing-related movement ease and spatial comfort. |
| **information/organization cues** | | |
| Organization of presented materials |  | Relevant to judgments of organizational clarity and logical viewing sequence. |
| Display structure |  | Relevant to judgments of viewing clarity for the presented materials. |
| Spatial arrangement of display elements |  | Relevant to judgments of spatial organization and viewing appropriateness. |
| **visual-atmospheric cues** | | |
| Lighting position and direction |  | Relevant to judgments of directional visual support and emphasis. |
| Illumination pattern |  | Relevant to judgments of brightness adequacy and visual uniformity. |
| Overall lighting quality |  | Relevant to judgments of visual readability and atmospheric clarity. |
| **service-usability cues** | | |
| Directional/information signage |  | Relevant to judgments of wayfinding support and information usability. |
| Main service point |  | Relevant to judgments of service visibility and accessibility. |
| Essential service facilities |  | Relevant to judgments of basic facility usability and user comfort. |
| **access/safety cues** | | |
| Step-free or assisted access features |  | Relevant to judgments of mobility-related access support. |
| Accessibility-related information | Not present in the setting; no separate image presented in the original questionnaire | Relevant to judgments of whether supportive access information was available. |
| Safety-related environmental arrangement |  | Relevant to judgments of safe use, including for more vulnerable users. |

***Note.*** The images reproduced in Tables S1–S2 are the visual stimuli presented to respondents in the original questionnaire for the corresponding appraisal judgments. They are reproduced here to document the stimulus content available during the comparative rating task. The tables describe visible or explicitly communicated features of the presented materials only and should not be interpreted as evaluations of objective environmental performance, usability, or psychological effect. The designation “Not present in the setting” indicates that the corresponding environmental feature was absent from the focal setting; accordingly, no separate visual example was presented for that item–setting combination.

**Table S2.**

*Visual stimuli presented to respondents for standardized exposure condition A*

| Cue category | Presented feature | | Appraisal relevance |
| --- | --- | --- | --- |
| **movement-related cues** | | | |
| Entry area |  | Relevant to judgments of entry legibility and initial orientation. | |
| Main circulation path |  | Relevant to judgments of movement continuity and step-free navigation. | |
| Main viewing area |  | Relevant to judgments of movement clarity and spatial guidance within the setting. | |
| Rest area |  | Relevant to judgments of availability of resting or pausing support. | |
| Retail/browsing area |  | Relevant to judgments of ease of browsing and movement within the area. | |
| **information/organization cues** | | | |
| Organization of presented materials |  | Relevant to judgments of coherence and interpretability of the viewing order. | |
| Display structure |  | Relevant to judgments of clarity in viewing presented materials. | |
| Spatial arrangement of display elements |  | Relevant to judgments of organization and viewing accessibility. | |
| **visual-atmospheric cues** | | | |
| Lighting position and direction |  | Relevant to judgments of directional lighting support and visual emphasis. | |
| Illumination pattern |  | Relevant to judgments of perceived brightness and illumination consistency. | |
| Overall lighting quality |  | Relevant to judgments of visual readability and atmospheric quality. | |
| **service-usability cues** | | | |
| Directional/information signage |  | Relevant to judgments of clarity of wayfinding and informational cues. | |
| Main service point |  | Relevant to judgments of recognizability and accessibility of service support. | |
| Essential service facilities |  | Relevant to judgments of usability and basic service comfort. | |
| **access/safety cues** | | | |
| Step-free or assisted access features | Not present in the setting; no separate image presented in the original questionnaire | Relevant to judgments of mobility-related access support. | |
| Accessibility-related information | Not present in the setting; no separate image presented in the original questionnaire | Relevant to judgments of whether supportive access information was available. | |
| Safety-related environmental arrangement |  | Relevant to judgments of perceived safety in use of the environment. | |

***Note.*** The images reproduced in Tables S1–S2 are the visual stimuli presented to respondents in the original questionnaire for the corresponding appraisal judgments. They are reproduced here to document the stimulus content available during the comparative rating task. The tables describe visible or explicitly communicated features of the presented materials only and should not be interpreted as evaluations of objective environmental performance, usability, or psychological effect. The designation “Not present in the setting” indicates that the corresponding environmental feature was absent from the focal setting; accordingly, no separate visual example was presented for that item–setting combination.

Because the same respondents rated both contexts using the same 17 indicators, cross-context similarity in the prespecified loading patterns was characterized using Tucker’s congruence coefficients (φ) calculated from the separate context-specific CFAs. Higher values indicate greater similarity between corresponding loading vectors. These coefficients are descriptive and do not constitute multigroup measurement-invariance testing.

**Table S3.**

*Descriptive loading congruence across contexts for the theory-guided domain grouping*

| Factor | Tucker φ | RMSE (loading diff) | Max \|diff\| | n items |
| --- | --- | --- | --- | --- |
| C | 0.990 | 0.092 | 0.174 | 5 |
| H | 0.997 | 0.055 | 0.068 | 3 |
| I | 0.994 | 0.097 | 0.165 | 3 |
| M | 0.998 | 0.039 | 0.052 | 3 |
| E | 0.999 | 0.091 | 0.130 | 3 |

***Note.*** Tucker’s φ summarizes similarity between corresponding loading vectors from the two context-specific CFAs.

Congruence was high across factors (φ = 0.990–0.999), indicating similar loading patterns across the two context-specific CFAs.

Residual diagnostics for the three-way mixed model (Score ~ Context × Domain × Age Group with random intercepts and random slopes for Context by respondent) are shown in Figures S1–S2. Random-effects variance components and approximate marginal and conditional R² are summarized in Table S4.

**Table S4.**

*Random-effects variance components and approximate model R²*

| Var(intercept) | Var(slope) | Cov(int, slope) | Corr(int, slope) | Residual var | ICC (approx) | R² marginal | R² conditional |
| --- | --- | --- | --- | --- | --- | --- | --- |
| 0.239 | 0.021 | 0.020 | 0.275 | 0.257 | 0.512 | 0.075 | 0.549 |

Figure S1. Q-Q plot of residuals from the three-way linear mixed model.

Figure S2. Residuals versus fitted values from the three-way mixed-effects model.

As a robustness check, we re-estimated the key interaction effects using Gaussian GEE with exchangeable within-respondent correlation and sandwich (robust) standard errors. The Context × Domain and Context × Domain × Age Group interactions remained significant under the population-averaged GEE specification with robust standard errors (Table S5). Robust coefficient estimates are reported in Table S6.

**Table S5.**

*Robust (sandwich) Wald tests from Gaussian GEE (exchangeable working correlation).*

| Effect | Wald χ² | df | p |
| --- | --- | --- | --- |
| Context × Domain (GEE robust Wald) | 88.978 | 4 | < .001 |
| Context × Domain × AgeGroup (GEE robust Wald) | 47.482 | 16 | < .001 |

***Note.*** Wald tests are based on Gaussian generalized estimating equations with exchangeable within-respondent correlation and sandwich (robust) standard errors. p values are rounded to three decimals; values less than .001 are reported as < .001.

**Table S6.**

*Robust GEE estimates for the full Context × Domain × Age Group model*

| Term | Estimate | Robust SE | Wald | p |
| --- | --- | --- | --- | --- |
| (Intercept) | 1.946 | 0.028 | 4685.999 | < .001 |
| Context A | 0.088 | 0.019 | 21.961 | < .001 |
| Domain H | -0.027 | 0.020 | 1.786 | .181 |
| Domain I | 0.022 | 0.021 | 1.076 | .300 |
| Domain M | 0.003 | 0.020 | 0.029 | .865 |
| Domain E | 0.442 | 0.040 | 123.206 | < .001 |
| Age Group. L | 0.189 | 0.077 | 5.976 | .015 |
| Age Group. Q | 0.092 | 0.070 | 1.730 | .188 |
| Age Group. C | -0.006 | 0.056 | 0.012 | .912 |
| Age Group^4 | 0.147 | 0.047 | 9.666 | .002 |
| Context A × Domain H | 0.051 | 0.030 | 2.844 | .092 |
| Context A × Domain I | -0.011 | 0.029 | 0.144 | .705 |
| Context A × Domain M | 0.108 | 0.027 | 16.557 | < .001 |
| Context A × Domain E | -0.186 | 0.035 | 28.498 | < .001 |
| Context A × Age Group. L | 0.054 | 0.043 | 1.588 | .208 |
| Context A × Age Group. Q | 0.137 | 0.040 | 11.539 | < .001 |
| Context A × Age Group. C | -0.005 | 0.045 | 0.014 | .906 |
| Context A × Age Group^4 | 0.055 | 0.039 | 2.005 | .157 |
| Domain H × Age Group. L | 0.097 | 0.049 | 3.945 | .047 |
| Domain I × Age Group. L | -0.083 | 0.048 | 3.077 | .079 |
| Domain M × Age Group. L | -0.005 | 0.046 | 0.013 | .908 |
| Domain E × Age Group. L | 0.042 | 0.099 | 0.186 | .667 |
| Domain H × Age Group. Q | 0.117 | 0.046 | 6.413 | .011 |
| Domain I × Age Group. Q | 0.064 | 0.046 | 1.913 | .167 |
| Domain M × Age Group. Q | 0.010 | 0.044 | 0.053 | .819 |
| Domain E × Age Group. Q | 0.235 | 0.089 | 7.016 | .008 |
| Domain H × Age Group. C | 0.021 | 0.044 | 0.226 | .635 |
| Domain I × Age Group. C | 0.095 | 0.047 | 4.094 | .043 |
| Domain M × Age Group. C | 0.139 | 0.044 | 10.151 | .001 |
| Domain E × Age Group. C | -0.250 | 0.092 | 7.318 | .007 |
| Domain H × Age Group^4 | -0.019 | 0.041 | 0.215 | .643 |
| Domain I × Age Group^4 | 0.087 | 0.045 | 3.710 | .054 |
| Domain M × Age Group^4 | 0.072 | 0.042 | 2.964 | .085 |
| Domain E × Age Group^4 | -0.193 | 0.075 | 6.620 | .010 |
| Context A × Domain H × Age Group. L | -0.176 | 0.070 | 6.249 | .012 |
| Context A × Domain I × Age Group. L | -0.027 | 0.067 | 0.161 | .689 |
| Context A × Domain M × Age Group. L | 0.024 | 0.061 | 0.158 | .691 |
| Context A × Domain E × Age Group. L | 0.132 | 0.086 | 2.352 | .125 |
| Context A × Domain H × Age Group. Q | -0.095 | 0.067 | 2.004 | .157 |
| Context A × Domain I × Age Group. Q | -0.047 | 0.067 | 0.497 | .481 |
| Context A × Domain M × Age Group. Q | 0.170 | 0.059 | 8.329 | .004 |
| Context A × Domain E × Age Group. Q | -0.022 | 0.078 | 0.079 | .779 |
| Context A × Domain H × Age Group. C | -0.012 | 0.070 | 0.028 | .868 |
| Context A × Domain I × Age Group. C | -0.044 | 0.063 | 0.473 | .491 |
| Context A × Domain M × Age Group. C | -0.066 | 0.061 | 1.174 | .279 |
| Context A × Domain E × Age Group. C | 0.139 | 0.080 | 3.022 | .082 |
| Context A × Domain H × Age Group^4 | 0.014 | 0.064 | 0.048 | .826 |
| Context A × Domain I × Age Group^4 | -0.142 | 0.064 | 4.861 | .027 |
| Context A × Domain M × Age Group^4 | -0.020 | 0.057 | 0.123 | .726 |
| Context A × Domain E × Age Group^4 | 0.127 | 0.066 | 3.666 | .056 |

Note. Robust GEE estimates are reported for the full Context × Domain × Age Group model. The reference levels were Context B for Context and Domain C for Domain. Domain H, I, M, and E denote hierarchical information organization, illumination and visual atmosphere, maintenance and service usability, and environmental access and safety cues, respectively. Age Group. L, Age Group. Q, and Age Group. C denote the linear, quadratic, and cubic orthogonal polynomial contrasts for the ordered adult age groups; Age Group^4 denotes the fourth-order polynomial contrast. p values are rounded to three decimals; values less than .001 are reported as < .001.

**Table S7.**

*Country-based sensitivity analysis of within-domain context contrasts*

| Code | Domain | All Estimate (B − A) | All SE | All p | All Direction | South Korea Estimate (B − A) | South Korea SE | South Korea p | South Korea Direction | China Estimate (B − A) | China SE | China p | China Direction |
| --- | --- | --- | --- | --- | --- | --- | --- | --- | --- | --- | --- | --- | --- |
| C | Circulation and spatial ease | -0.082 | 0.026 | .002 | Context A higher | -0.075 | 0.030 | .013 | Context A higher | -0.101 | 0.052 | .053 | Context A higher |
| H | Hierarchical information organization | -0.164 | 0.026 | < .001 | Context A higher | -0.149 | 0.030 | < .001 | Context A higher | -0.204 | 0.052 | < .001 | Context A higher |
| I | Illumination and visual atmosphere | -0.067 | 0.026 | .010 | Context A higher | -0.056 | 0.030 | .062 | Context A higher | -0.097 | 0.052 | .065 | Context A higher |
| M | Maintenance and service usability | -0.187 | 0.026 | < .001 | Context A higher | -0.179 | 0.030 | < .001 | Context A higher | -0.207 | 0.052 | < .001 | Context A higher |
| E | Environmental access and safety cues | 0.122 | 0.026 | < .001 | Context B higher | 0.107 | 0.030 | < .001 | Context B higher | 0.163 | 0.052 | .002 | Context B higher |

Note. All planned contrasts were coded as Context B − Context A. Negative estimates indicate higher ratings for Context A, whereas positive estimates indicate higher ratings for Context B. The pattern is reported to show domain-specific contrast direction rather than overall context superiority.

**Table S8.**

*Standardized factor loadings from the context-specific CFA diagnostics for the theory-guided five-domain item grouping*

| Item | Factor | Std. loading |
| --- | --- | --- |
| **Context A** | | |
| i1 | C | 0.705 |
| i2 | C | 0.609 |
| i3 | C | 0.601 |
| i4 | C | 0.603 |
| i5 | C | 0.467 |
| i6 | H | 0.588 |
| i7 | H | 0.748 |
| i8 | H | 0.707 |
| i9 | I | 0.612 |
| i10 | I | 0.692 |
| i11 | I | 0.565 |
| i12 | M | 0.611 |
| i13 | M | 0.647 |
| i14 | M | 0.656 |
| i15 | E | 0.661 |
| i16 | E | 0.599 |
| i17 | E | 0.716 |
| **Context B** | | |
| i1 | C | 0.606 |
| i2 | C | 0.615 |
| i3 | C | 0.627 |
| i4 | C | 0.642 |
| i5 | C | 0.641 |
| i6 | H | 0.634 |
| i7 | H | 0.680 |
| i8 | H | 0.660 |
| i9 | I | 0.644 |
| i10 | I | 0.695 |
| i11 | I | 0.730 |
| i12 | M | 0.663 |
| i13 | M | 0.671 |
| i14 | M | 0.621 |
| i15 | E | 0.710 |
| i16 | E | 0.729 |
| i17 | E | 0.792 |

**Table S9.**

*Model-based within-domain context contrasts (Context B − Context A) by age group*

| Age group | Domain | Estimate | SE | 95% CI | z | p | BH-FDR adjusted p |
| --- | --- | --- | --- | --- | --- | --- | --- |
| 20–29 | C | -0.135 | 0.046 | [-0.224, -0.046] | -2.962 | .003 | .004 |
| 20–29 | H | -0.252 | 0.046 | [-0.341, -0.163] | -5.532 | < .001 | < .001 |
| 20–29 | I | -0.112 | 0.046 | [-0.202, -0.023] | -2.468 | .014 | .014 |
| 20–29 | M | -0.337 | 0.046 | [-0.426, -0.248] | -7.405 | < .001 | < .001 |
| 20–29 | E | 0.174 | 0.046 | [0.085, 0.264] | 3.830 | < .001 | < .001 |
| 30–39 | C | -0.004 | 0.060 | [-0.122, 0.114] | -0.068 | .946 | .946 |
| 30–39 | H | -0.122 | 0.060 | [-0.241, -0.004] | -2.030 | .042 | .106 |
| 30–39 | I | -0.054 | 0.060 | [-0.173, 0.064] | -0.902 | .367 | .612 |
| 30–39 | M | -0.027 | 0.060 | [-0.145, 0.091] | -0.451 | .652 | .815 |
| 30–39 | E | 0.190 | 0.060 | [0.072, 0.309] | 3.157 | .002 | .008 |
| 40–49 | C | -0.054 | 0.050 | [-0.153, 0.044] | -1.076 | .282 | .470 |
| 40–49 | H | -0.167 | 0.051 | [-0.267, -0.068] | -3.302 | < .001 | .005 |
| 40–49 | I | 0.033 | 0.051 | [-0.065, 0.132] | 0.660 | .509 | .571 |
| 40–49 | M | -0.057 | 0.051 | [-0.156, 0.042] | -1.132 | .258 | .470 |
| 40–49 | E | 0.029 | 0.051 | [-0.071, 0.128] | 0.566 | .571 | .571 |
| 50–59 | C | -0.045 | 0.067 | [-0.176, 0.086] | -0.674 | .500 | .500 |
| 50–59 | H | -0.067 | 0.067 | [-0.198, 0.065] | -0.998 | .318 | .398 |
| 50–59 | I | -0.133 | 0.067 | [-0.265, -0.002] | -1.997 | .046 | .076 |
| 50–59 | M | -0.167 | 0.067 | [-0.299, -0.036] | -2.496 | .013 | .031 |
| 50–59 | E | 0.242 | 0.067 | [0.111, 0.373] | 3.619 | < .001 | .001 |
| 60+ | C | -0.200 | 0.088 | [-0.372, -0.027] | -2.271 | .023 | .058 |
| 60+ | H | -0.087 | 0.088 | [-0.259, 0.086] | -0.988 | .323 | .323 |
| 60+ | I | -0.116 | 0.088 | [-0.289, 0.057] | -1.317 | .188 | .235 |
| 60+ | M | -0.391 | 0.088 | [-0.564, -0.219] | -4.444 | < .001 | < .001 |
| 60+ | E | -0.145 | 0.088 | [-0.318, 0.028] | -1.646 | .100 | .166 |

Note. Values are model-based contrasts from the three-way mixed-effects model and were coded as Context B − Context A. Positive values indicate higher ratings for Context B, whereas negative values indicate higher ratings for Context A.

Figure S3. Age-Stratified Within-Domain Contrasts between Contexts

***Note.*** Cells show age-stratified model-based contrasts (Δ = Context B − Context A). Negative values indicate higher ratings for Context A, whereas positive values indicate higher ratings for Context B. Asterisks indicate BH-FDR-adjusted p < .05.

**Table S10.**

*Country-adjusted sensitivity tests for the primary mixed-effects models*

| Test | LL full | LL reduced | LR χ² | df | p |
| --- | --- | --- | --- | --- | --- |
| Context × Domain | −7100.172 | −7144.990 | 89.637 | 4 | < .001 |
| Context × Domain × Age Group | −6982.846 | −7003.712 | 41.731 | 16 | < .001 |

Note. Participant country was included as a between-subject fixed-effect covariate in both the full and reduced models. The original respondent-specific random-intercept and Context-slope structure was retained, and no Country interaction terms were introduced. The country-adjusted analyses yielded the same inferential conclusions as the corresponding unadjusted models.

**Table S11.**

*Repeated age-balanced subsampling sensitivity analysis*

| Outcome | Full N = 804 | Age-balanced N = 345, 200 iterations | Stability |
| --- | --- | --- | --- |
| Context × Domain | LR χ² = 89.637 | Median LR χ² = 32.236 [IQR 27.214, 36.839] | 200/200 (100%) p < .05 |
| Context × Domain × Age Group | LR χ² = 41.731 | Median LR χ² = 26.313 [IQR 21.459, 30.251] | 101/200 (50.5%) p < .05 |
| C contrast | −0.082 | −0.0858 [−0.0980, −0.0742] | 100% same sign |
| H contrast | −0.164 | −0.1367 [−0.1539, −0.1208] | 100% same sign |
| I contrast | −0.067 | −0.0754 [−0.0957, −0.0580] | 100% same sign |
| M contrast | −0.187 | −0.1937 [−0.2155, −0.1768] | 100% same sign |
| E contrast | +0.122 | +0.0986 [+0.0819, +0.1140] | 100% same sign |

Note. In each of 200 iterations, 69 respondents were sampled without replacement from each of the five age groups, yielding N = 345. All repeated observations for selected respondents were retained, and the primary mixed-effects model specification was unchanged. Because the 60+ group contained exactly 69 respondents, all members of that group were retained in every iteration. For the interaction tests, stability indicates the proportion of iterations with p < .05; for the domain contrasts, stability indicates the proportion retaining the same sign as the full N = 804 analysis. No convergence or singular-fit failures occurred.
